# Recombinant Platelet-Derived Growth Factor-BB (rhPDGF-BB) Augmented Collagen Grafting for Oral Mucosal Defect Reconstruction

**DOI:** 10.1007/s12663-024-02194-5

**Published:** 2024-05-10

**Authors:** Kshithi Kudlu, Haneesh Amit Domah, Fayis Mohammed Anwar, Vijendra S. Shenoy, Vikrant Kamboj, Neehal Zuturu

**Affiliations:** Department of ENT and Head & Neck Surgery, Kasturba Medical College, Mangalore, Manipal Academy of Higher Education, Manipal, India

**Keywords:** Collagen, Contracture, Oral cavity, Becaplermin, Reconstruction

## Abstract

**Objective:**

To assess the suitability of collagen grafting and compare the outcomes between rhPDGF-BB (recombinant human platelet-derived growth factor-BB) augmented collagen grafting and collagen alone grafting for oral mucosal defect reconstruction.

**Method:**

A total of 30 patients, who were planned for surgical excision of various intraoral lesions, participated in the study. These participants were then randomly assigned into two groups. Group I underwent reconstruction with collagen sheet with rhPDGF-BB, while group II underwent reconstruction with collagen sheet alone. Post-operatively, haemostasis, pain, granulation, epithelialisation and healing contracture were assessed.

**Results:**

The lesions that were treated included leucoplakia, erythroplakia, oral submucous fibrosis and verrucous carcinoma. Use of collagen was associated with good haemostasis, pain relief, granulation tissue formation and epithelialisation. Group I was found to develop slightly better granulation tissue, epithelisation and lesser degree of tissue contracture compared to group II, although these results were not significant statistically (*p* value = 0.464, 0.705 and 1.000 respectively).

**Conclusion:**

This study shows that use of collagen graft augmented with rhPDGF-BB could be beneficial in oral cavity reconstruction following resection of small lesions, although further research with larger sample size is proposed to confirm the benefit.

## Introduction

Adequate reconstruction of surgical defects of the oral cavity following therapeutic or diagnostic resection of lesions is a challenging aspect of surgery. Reconstruction is affected by several factors. Collagen sheets have been used in previous studies as material for reconstruction of oral cavity defects. Advantages are that collagen acts as a drug carrier as well as a biological dressing, its ease of availability, good oral tissue tolerance, convenience of application, minimal adverse effects, obviation of need for another surgery for graft harvest or detachment of pedicle, no additional morbidity and no problems related to donor site healing [1–4].

Becaplermin is human platelet-derived growth factor also called platelet-derived growth factor-BB has been used in conjunction with good local care for the management of lower extremity diabetic neuropathic ulcers. This is commercially available as Plermin 0.01% gel containing becaplermin (100 mcg). In periodontology, this has been studied widely among the growth factors. Its efficacy has been demonstrated in both soft and hard tissue regeneration in studies over the last few decades. After tissue injury, blood platelets release PDGF, where they bind to specific cell surface receptors. As a consequence, the process of wound healing is enhanced through chemotaxis and mitogenesis [5, 6].

Considering the beneficial effects of collagen and rhPDF-BB in the healing of oral mucosal defects, we conducted our study to assess the suitability of using collagen sheet augmented with rhPDGF-BB for oral mucosal defect reconstruction and also to compare its surgical outcome with collagen grafting alone.

## Materials and Methods

This was an observational study conducted at a tertiary referral centre. Participants planned for surgical excision of various intraoral lesions like patches of leucoplakia, erythroplakia, oral submucosal fibrosis, mucosal hyperpigmentation and verrucous carcinoma with surgical defects ranging from 30 to 50 mm were included. Patients who had chronic and debilitating diseases and those with defects larger than 50 mm were excluded from this study. After obtaining a detailed history, a thorough clinical examination was conducted. Findings of ulcerative lesion, whitish/reddish patches, restricted mouth opening, palpable fibrous bands, etc. were recorded in standard format. Informed consent was obtained from every patient. Approval of the ethical committee of institutional review board was obtained.

After enrolling into study, the patients were randomly assigned to undergo either reconstruction with collagen sheet along with rhPDGF-BB or collagen sheet alone. All surgical excisions were performed under general anaesthesia. After excision of lesion, the defect was reconstructed using collagen sheet which was sutured to the edges of the defect using 3-0 vicryl. In the patients assigned for receiving rhPDFG-BB, the gel was applied over the area after reconstruction.

Depending upon the procedure undertaken, patients were categorised into groups of 15 members each as follows:

Group I: Patients who underwent reconstruction with collagen sheet and rhPDGF-BB over the mucosal defect.

Group II: Reconstruction with collagen sheet alone over the mucosal defect.

During the post-operative period, povidone-iodine mouth wash as oral rinse thrice daily, as well as physiotherapy was followed by all patients.

Parameters evaluated were:

1. Haemostasis achieved by the membrane. This was evaluated intraoperatively and scored using following scale: (a) good—no bleeding or haemostasis achieved within 5 min, (b) fair—haemostasis achieved after more than 5 min but no interventions required and (c) poor—bleeding requiring further intervention for haemostasis
2. Pain was assessed on day 7 after surgery. This was assessed according to the patient’s perception of pain as: (a) good (none to mild), (b) fair (moderate) and (c) poor (severe).
3. Appearance of granulation tissue at completion of two weeks as: (a) good (entire wound), (b) fair (more than half of the wound) and (c) poor (less than half of the wound).
4. Epithelialisation—This was evaluated a month after the procedure: (a) good (entire wound), (b) fair (more than half of the wound) and (c) poor (less than half of the wound).
5. Wound contracture. This was also assessed after a month: (a) good (< 25%), (b) fair (25–50%) and (c) poor (severe > 50%).

Statistical analysis—The data collected were coded and entered in IBM SPSS Statistics for Windows, version 25.0. Results were expressed as proportions using appropriate tables and figures. Chi-square test was used for comparison across groups. A *p* value of < 0.05 was considered statistically significant.

## Results

A total of 30 patients were included in the study. The patients belonged to age groups ranging from 31 to 78 years with maximum patients in 41–60 years group (56.7%). Of the 30 patients, 12 were female and 18 were male with equal distribution in both groups of 6 females and 9 males. There were 12 cases of leucoplakia, 6 cases of erythroplakia, 8 cases of oral submucosal fibrosis and 4 cases of verrucous carcinoma.

**Fig. 1:**
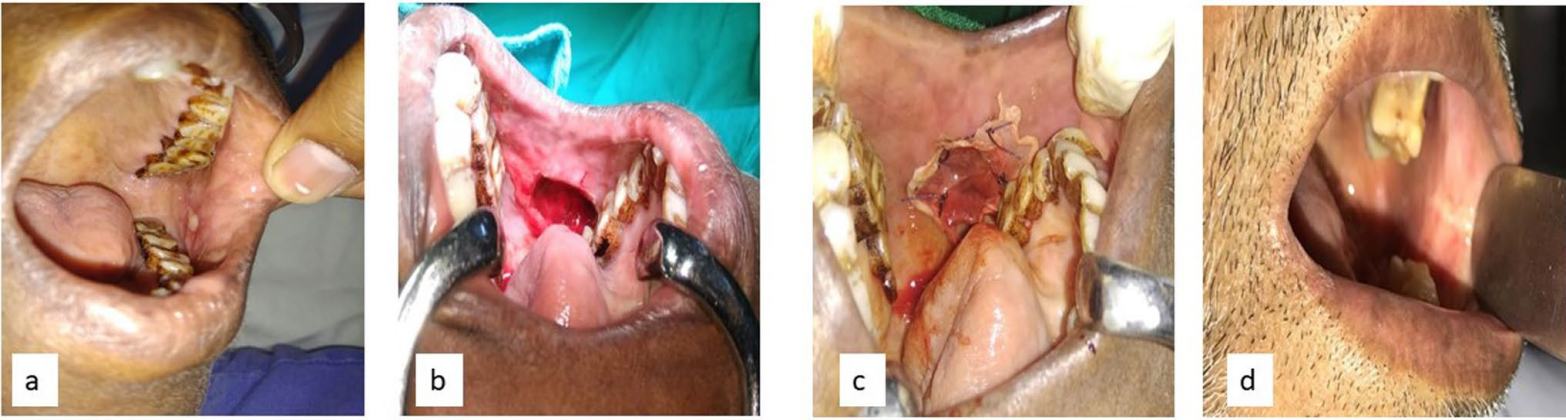
Preoperative, intraoperative and post-operative images

Figure 1 demonstrates a mucosal lesion that was excised following which collagen reconstruction with rhPDGF-BB was undertaken and the healed defect after 4 months.

### Haemostasis

Out of 15 patients in each group, 13 in group I and 12 in group II had good.

Haemostasis and others had fair haemostasis during the surgery. None of the cases required additional intervention to control intraoperative bleeding.

There was no significant difference found between the two groups with *p* = 1.000.

### Pain

At the end of 7^th^ post-operative day, in each group, out of 15 patients, nine patients had good pain relief and six patients had fair pain relief.

**Fig. 2:**
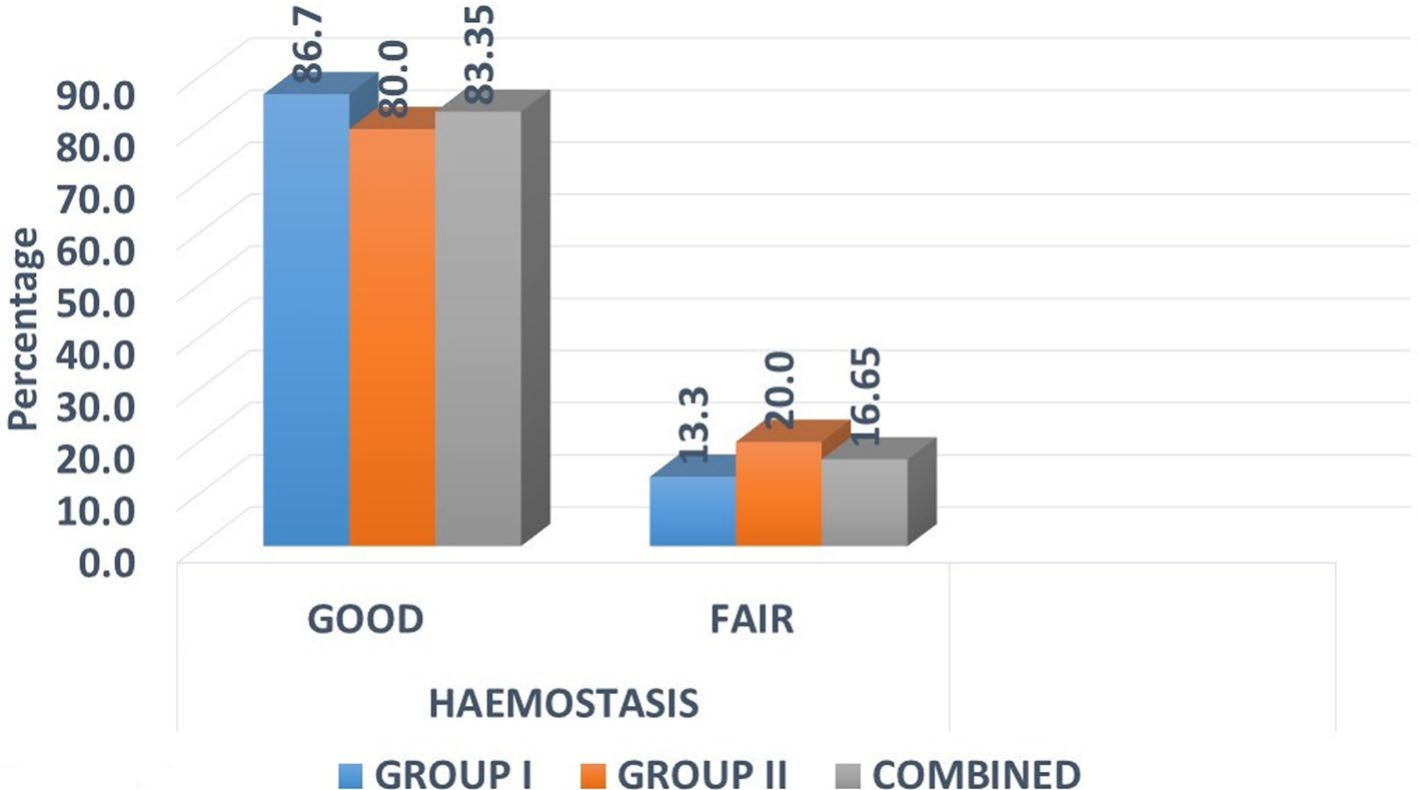
Distribution of samples by haemostasis

There was no significant difference found between the two groups with *p* = 1.000 (Fig. 2).

### Granulation

This was assessed at 2 weeks after surgery. Out of 15 patients in each group, nine in group I and seven in group II had good granulation formation and the rest had fair granulation formation.

**Fig. 3:**
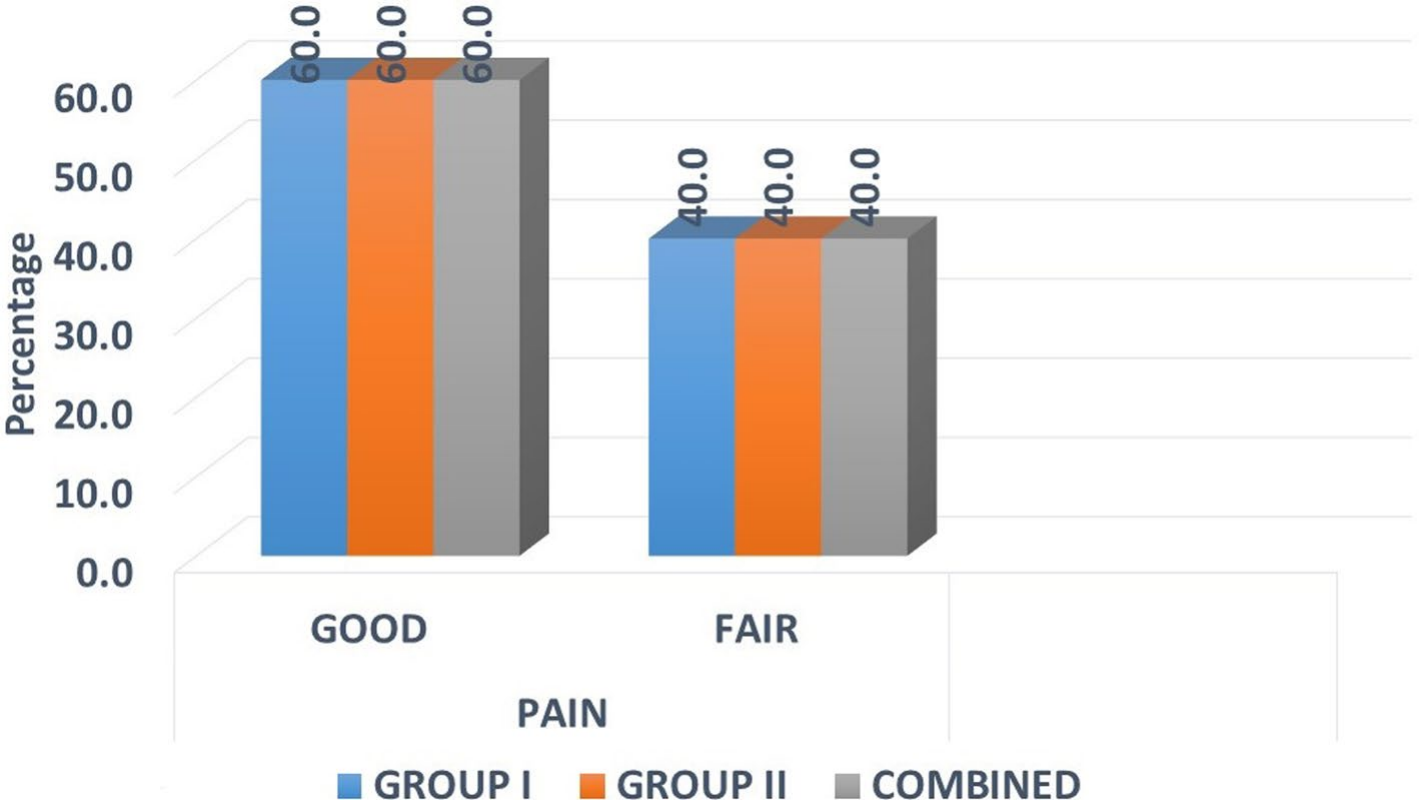
Distribution of samples by pain score

There was no significant difference found between the two groups with *p* = 0.464 (Fig. 3).

### Epitheialisation

**Fig. 4:**
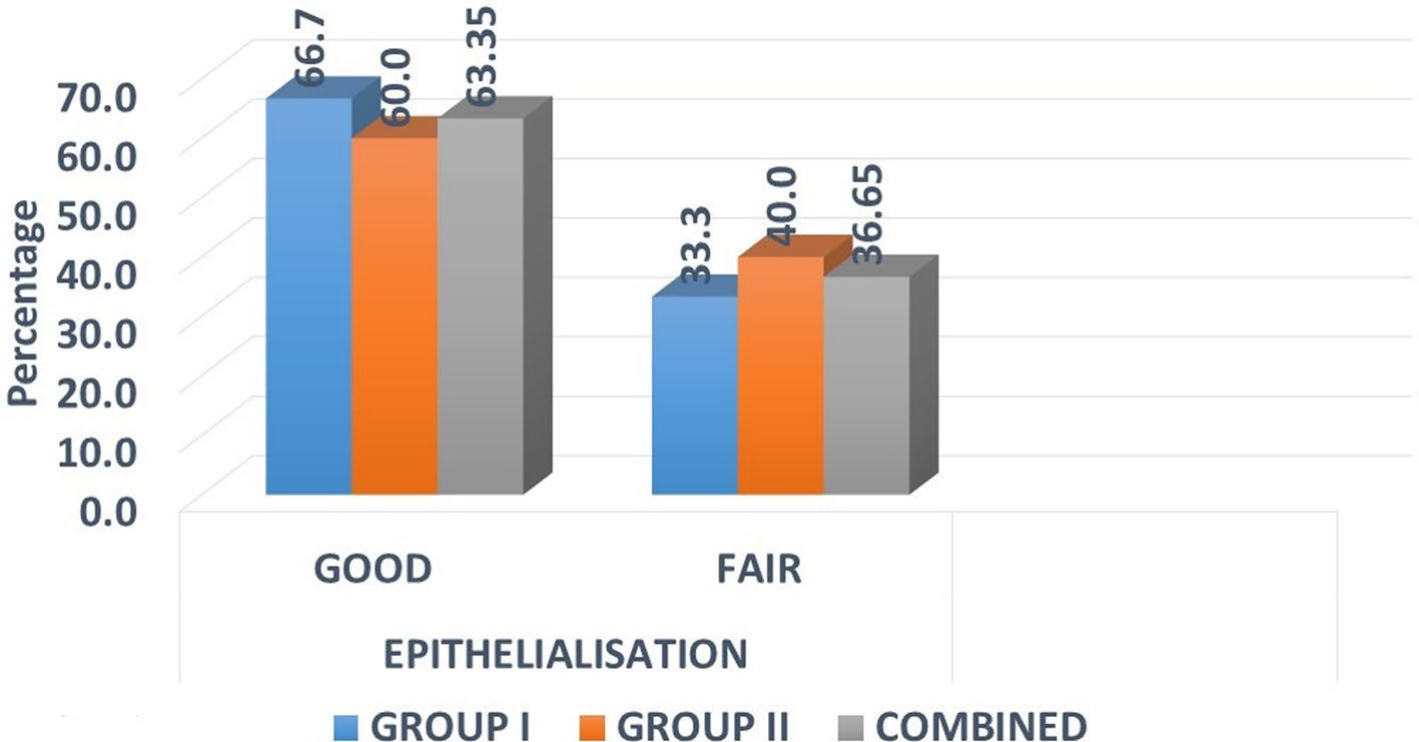
Distribution of samples by epithelialisation

At 4 weeks post-operatively, out of 15 patients in group I, good epithelialisation was seen in 10 patients, whereas in group II, out of 15 patients, nine had good epithelialisation and others had fair epithelialisation. Epithelialisation was better in group I who underwent collagen reconstruction with rhPDGF-BB, but it was statistically not significant with *p* = 0.705 (Fig. 4).

### Contracture

**Fig. 5:**
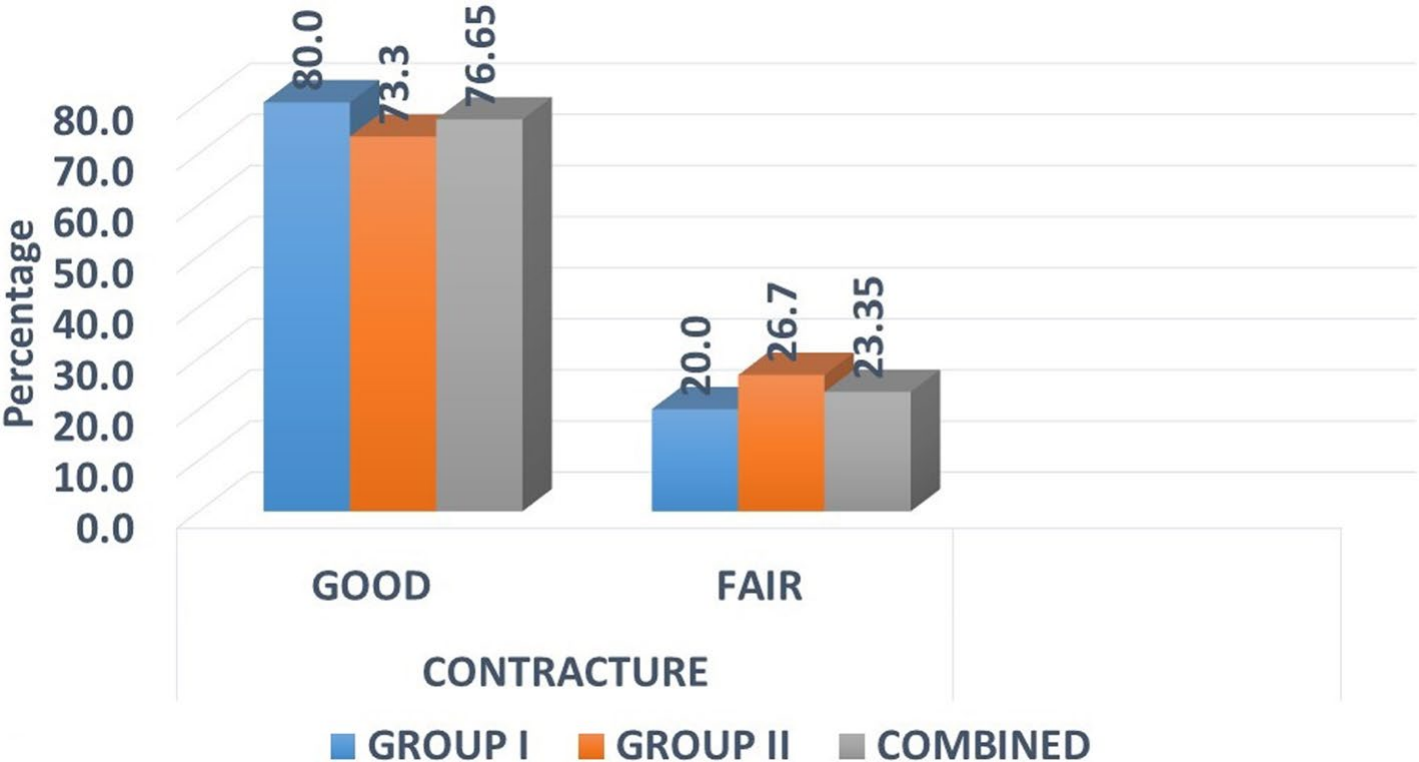
Distribution of samples by contracture

At 4 weeks post-operatively, wound contracture was evaluated. Good contracture, i.e. < 25%, was seen in 12 patients in group I and 11 in the group II. Others had fair contracture. There was no significant difference found between the two groups with *p* = 1.000 (Fig. 5).

## Discussion

The wounds of the oral environment are regularly contaminated with saliva as well as food, exacerbated by movements of cheek and tongue. Previous studies have reported that frequency of infection and contracture degree are extensively decreased when wounds are covered as opposed to left uncovered. Studies have reported that dressings make a physiological interface between wound surface and environment, thus permitting repair [2, 7].

Efforts are continuously being made to develop newer treatment methods that are less invasive with minimal side effects. Our study evaluated two modalities in reconstruction of oral cavity post-operative defects: collagen, used as reconstruction material to cover the surgical wound, and platelet-derived growth factor which enhances wound healing. As an alternative material to mucosal or skin grafts, collagen can be used to cover surgical defects intraorally. Since collagen is an actual component of the skin, it has beneficial effect throughout the wound healing process. As compared to tissue grafts, collagen membrane is easily available commercially and hence obviates the need for a second surgery for graft harvesting and its associated morbidity [2, 3, 8].

Many studies have focused on methods to improve healing in oral cavity defects, and there is an increasing interest in growth factor to aid this process. PDGF-BB is a blood product containing a lot of growth factors which do not have side effects. It helps in cell proliferation, differentiation, chemotaxis and matrix synthesis. Its effects are important in the initial phases of healing and tissue regeneration [5, 6].

Collagen is a very efficient haemostatic agent. The platelets adhere to collagen, followed by swelling and release of substances that initiate haemostasis. Our study noted good haemostasis in 25 out of 30 (83.3%) patients and fair in five patients (16.7%) [9].

Collagen provides coverage for open nerve endings and hence diminishes the intensity of pain when used over raw wounds. However, some degree of pain can be present post-surgery which is usually attributable to post-surgical traumatic inflammation. Our study showed good pain relief in 18 cases (60%) and fair in 12 cases (40%) [3].

Although collagen can undergo collagenolysis, collagen membranes are robust enough to allow formation of granulation tissue, which appeared uniform and clinically healthy. Our study showed good granulation formation in 16 cases (53.35%) and fair in 14 cases (46.65%) two weeks post-operatively [2, 10].

Collagen triggers the adhesiveness of platelets and stimulates the release phenomenon, leading to aggregation of nearby platelets. The collagen membrane can achieve early epithelisation. The same was noted in our study with good epithelialisation in 19 cases (63.35%) and fair in 11 cases (36.65%) [11].

Scar contracture can be prevented by controlling infection and accelerated re-epithelialisation of wound, and it can be fulfilled with the help of collagen and rhPDGF-BB. In our study, we had with 23 cases with good contracture and 7 with fair contracture.

We also tried to assess the role of rhPDGF-BB when used along with collagen grafting. We noticed that patients in group I who underwent collagen grafting along with rnPDGF had better granulation tissue response, better epithelialisation and lesser tissue contracture in comparison with group II, although this was not statistically significant (*p* value = 0.464, 0.705 and 1.000, respectively). Haemostasis and post-operative pain were similar among both the groups.

The use of collagen as a wound dressing material was shown to be benefical in all aspects of wound healing in our study similar to what has been noted in previous studies. Although augmentation with rhPDGF was associated with better granulation tissue response, epithelialisation and lesser scar contracture, the results were not statistically significant compared to grafting with collagen alone. This could probably be attributed to the relatively small sample size, and hence, the authors recommend further studies in larger size sample to observe the effects.

## Conclusions

Collagen sheet is a suitable alternative for reconstruction of small oral cavity defects that is not associated with additional morbidity of tissue graft and donor site complications. This study shows there could be an enhanced benefit of using collagen graft augmented with rhPDGF-BB in oral cavity reconstruction over collagen alone.
